# TIRF imaging of Fc gamma receptor microclusters dynamics and signaling on macrophages during frustrated phagocytosis

**DOI:** 10.1186/s12865-016-0143-2

**Published:** 2016-03-12

**Authors:** Jia Lin, Svetlana Kurilova, Brandon L. Scott, Elizabeth Bosworth, Bradley E. Iverson, Elizabeth M. Bailey, Adam D. Hoppe

**Affiliations:** Department of Chemistry and Biochemistry, Avera Health and Science Center 131, South Dakota State University, Brookings, SD 57007 USA; Department of Pathology, University of New Mexico Health Sciences Center, University of New Mexico, MSC 08–4640, Albuquerque, New Mexico 87131 USA; BioSNTR, South Dakota State University, Brookings, SD 57007 USA

**Keywords:** Fcγ receptor, IgG, TIRF, frustrated phagocytosis, receptor synapses, macrophage

## Abstract

**Background:**

Recent evidence indicates that in addition to the T-cell receptor, microclustering is an important mechanism for the activation of the B-cell receptor and the mast cell Fcε-receptor. In macrophages and neutrophils, particles opsonized with immunoglobulin G (IgG) antibodies activate the phagocytic Fcγ-receptor (FcγR) leading to rearrangements of the actin cytoskeleton. The purpose of this study was to establish a system for high-resolution imaging of FcγR microclustering dynamics and the recruitment of the downstream signaling machinery to these microclusters.

**Methods:**

We developed a supported lipid bilayer platform with incorporated antibodies on its surface to study the formation and maturation of FcγR signaling complexes in macrophages. Time-lapse multicolor total internal reflection microscopy was used to capture the formation of FcγR-IgG microclusters and their assembly into signaling complexes on the plasma membrane of murine bone marrow derived macrophages.

**Results:**

Upon antibody binding, macrophages formed FcγR-IgG complexes at the leading edge of advancing pseudopods. These complexes then moved toward the center of the cell to form a structure reminiscent of the supramolecular complex observed in the T-cell/antigen presenting cell immune synapse. Colocalization of signaling protein Syk with nascent clusters of antibodies indicated that phosphorylated receptor complexes underwent maturation as they trafficked toward the center of the cell. Additionally, imaging of fluorescent BtkPH domains indicated that 3′-phosphoinositides propagated laterally away from the FcγR microclusters.

**Conclusion:**

We demonstrate that surface-associated but mobile IgG induces the formation of FcγR microclusters at the pseudopod leading edge. These clusters recruit Syk and drive the production of diffusing PI(3,4,5)P_3_ that is coordinated with lamellar actin polymerization. Upon reaching maximal extension, FcγR microclusters depart from the leading edge and are transported to the center of the cellular contact region to form a synapse-like structure, analogous to the process observed for T-cell receptors.

**Electronic supplementary material:**

The online version of this article (doi:10.1186/s12865-016-0143-2) contains supplementary material, which is available to authorized users.

## Background

**Fig. 1:**
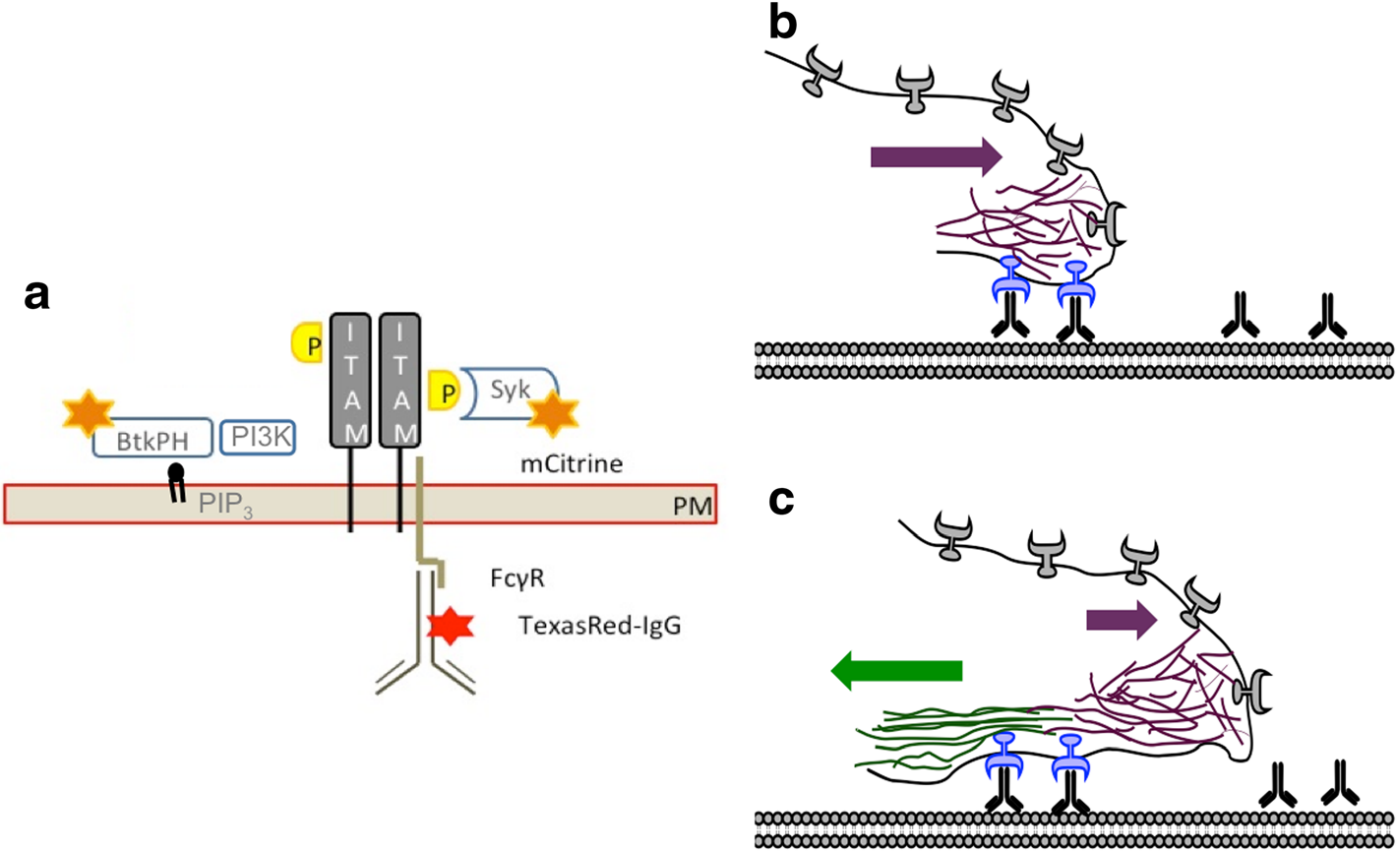
Model of FcγR signaling and microclustering relative to actin and pseudopod extension. **a** FcγR ligation by IgG drives phosphorylation of ITAMs and subsequent recruitment of signaling proteins including Syk and PI3K. Fluorescent labeling of IgG (red star) allowed observation of IgG-FcγR complexes relative to the recruitment of Syk or BtkPH (gold stars) which specifically binds the PI3K product, PI(3,4,5)P_3_. **b-c** Frustrated phagocytosis in response to IgG-presented on a supported lipid bilayer is shown schematically. The zipper model, which describes macrophage engulfment of IgG-coated particles, suggests FcγR-IgG interaction occurs through sequential engagement of new receptors during the advancement of the phagosome. Activated FcγRs (blue) cluster and are initially driven forward by polymerizing actin (purple, **b**). These complexes then disengage from the polymerizing actin and move toward the center of the cell by attaching to retrograde actin (green, **c**). New FcγR complexes form as new FcγR bind IgG at the leading edge (grey). The arrows indicate the direction of the polymerizing actin, retrograde actin and the associated FcγR cluster movement

Macrophages phagocytize bacteria and viruses that are opsonized by immunoglobulin G (IgG) following activation of Fcγ receptors (FcγR). FcγR clustering is required for the phosphorylation of Immunoreceptor Tyrosine-Based Activation Motifs (ITAMs) in the FcγR cytoplasmic tail (FcγRIIa) and associated transmembrane adaptors such as the common gamma-chain for FcγR I and III leading to the recruitment and activation of Syk kinase (Fig. 1a) [1–5]. Syk-mediated phosphorylation in-turn, drives remodeling of the actin cytoskeleton activating numerous downstream pathways including Rho-family GTPases and phosphatidylinositol 3-kinase (PI3K) to coordinate the phagocytosis process and transcriptional activation of inflammatory pathways [4, 6]. FcγR-mediated phagocytosis typically occurs via zippering mechanism, in which newly ligated FcγR guides cell membranes over the opsonized particle [4, 7–10]. In this model, FcγR-IgG signaling complexes drive extension of the pseudopod over the particle as new receptors are activated, at the leading edge, and then deactivated as the membrane advances (Fig. 1b-c) [11, 12]. FcγR-IgG signaling complexes must coordinate the formation of the phagosome through the action of second messengers such as PI3K-mediated phosphorylation of the 3′ position of PI(4,5)P_2_ (phosphatidylinositol 4,5-bisphosphate) to produce PI(3,4,5)P_3_ (phosphatidylinositol (3,4,5)-trisphosphate) [4, 13–17]. Locally synthesized PI(3,4,5)P_3_ recruits numerous downstream signaling molecules that shape the plasma membrane into the phagocytic cup [4, 9, 18]. Elevated PI(3,4,5)P_3_ concentration persists until closure of the phagosome while also increasing the activity of GEFs (guanine nucleotide exchange factor) for small GTPases. Thus, existing models for FcγR signaling predict three signaling stages for the receptor: FcγR clustering for activation, initiation of actin-driven protrusion (early signals) and late signals associated with phagosome closure and identity.

Of the immunoreceptors, microclustering of the T cell receptor (TCR) is the most studied for its role in forming an immunological synapse (IS) during interaction with antigen-presenting cells (APC) [9]. Actin rearrangements downstream of the TCR drive the formation of the IS and it’s bull’s eye arrangement known as the supramolecular activation cluster (SMAC). After receptor ligation, ZAP70 or Syk are recruited to the TCR microclusters where they mediate the phosphorylation of downstream signaling molecules [19]. Formation of the IS then mediated by F-actin rich protrusions that move around the distal SMAC (dSMAC, actin rich region) in a radial wave. TCR microclusters migrate inward along with downstream signaling molecules, such as Syk, Lyn, and VAV1, forming the central SMAC (cSMAC) [9, 20–24]. Similarly to TCRs, the B cell receptor (BCR) undergoes microclustering following binding with antigen triggers [22]. Recently, FcεRs on mast cells have been observed to form microclusters upon contact with lipid bilayer presenting IgE. In all cases, these microclusters were directly transported to the center of the cell to form a patch [25, 26]. Together, these studies indicate that microclustering is a common theme for immunoreceptors.

Here, we captured the clustering behavior of FcγR-IgG complexes in macrophages using Total Internal Reflection Fluorescence (TIRF) Microscopy [27–29]. The ‘evanescent field’ generated by TIRF selectively excites fluorophores within 200 nm above the glass surface, thereby reducing out-of-focus fluorescence from remainder of the cell. By taking advantage of a supported lipid bilayer (SLB) to present IgG, TIRF microscopy can provide high-resolution imaging of the FcγR microcluster dynamics during initial macrophage interactions with the surface followed by frustrated phagocytosis. Furthermore, we applied this system to capture the dynamic recruitment of downstream signaling proteins to FcγR-IgG microclusters by expressing them as fluorescent protein fusions. These data provide a framework for understanding the transitions in signaling states of FcγRs during actin polymerization and phagocytosis in macrophages.

## Methods

### Materials

Alexa Fluor 594 IgG Fraction Monoclonal Mouse Anti-Biotin (Code: 200-582-211) was from Jackson ImmunoResearch Inc. l-Palmitoyl-2-oleoyl-sn-glycero-3-phosphocholine (POPC) and 1-oleoyl-2-(12-biotinyl(aminododecanoyl))-*sn*-glycero-3-phosphoethanolamine (Biotin-PE) were ordered from Avanti Polar Lipids. Sulfuric acid (H_2_SO_4_), hydrogen peroxide (H_2_O_2_), chloroform and glucose were from Sigma Aldrich. Dulbecco’s Modified Eagle Medium (DMEM) was obtained from Cellgro (Manassas, VA). Phosphate buffered saline (PBS) and DiI were purchased from Thermo Fisher Scientific Inc. Fetal Bovine Serum (FBS) was from Atlanta Biologicals (Flowery Branch, GA). Anti-Biotin was purchased from Neomarkers (Fremont, CA). Platinum-E Retroviral Packaging Cell Line (Plat-E) were purchased from Cellbiolabs, San Diego, CA. FuGENE transfection reagent was obtained from Roche Applied Science (Germany). All supplementary materials were of the highest grade commercially available.

### Glass supported lipid bilayer

The SLB was formed by spontaneous fusion of lipid vesicles. To achieve this, Biotin-PE and POPC were mixed at a molar ratio of 1:100 with total lipid concentration of 400 μg/ml. The lipid mixture was then dissolved in chloroform and dried under air for 10 min. The lipid film was re-suspended in PBS containing 2 mM Mg^2+^. The obtained solution was sonicated for 5 min using a probe sonicator (Branson Ultrasonics, Danbury, CT). Bilayer was formed on Piranha acid (H_2_SO_4_ (30 %, *v/v*):H_2_O_2_ (3:1, *v/v*)) cleaned coverslip by incubation in a water bath at 37 °C for 15 min. Excess liposomes were exchanged with imaging buffer (PBS + 5 mM glucose). The bilayer coated coverslip was kept in a buffer solution during washing and transferring to imaging chamber to protect SLB from drying out and to keep it uniform [25]. Alexa Fluor 594 succidiminal ester was conjugated to anti-Biotin IgG for antibody fluorescent labeling (Jackson ImmunoResearch Inc.). The labeled antibody was incubated with SLB at 37 °C for 30 min. Excess IgG was washed with imaging buffer.

The mobility of SLB was confirmed by fluorescence recovery after photobleaching (FRAP) microscopy (Additional file 1). Briefly, the mobility of SLB labeled with Bodipy (5 μg/ml for 5 min) was observed by photobleaching the area of SLB and then imaging recovery of the fluorescent signal at the bleached location [25, 26].

### Cell culture and retroviral transduction of signaling proteins

Murine bone marrow derived macrophages (BMM) were obtained as described in [30]. Bone marrow was extruded from femurs and tibia of B57/BL6 mice (Charles River Laboratories, Wilmington, MA). The marrow was cultured in DMEM media containing 30 % L-cell supernatant as a source of MCSF (macrophage colony-stimulating factor), 20 % heat-inactive FBS. Cells were supplemented with fresh media to continue differentiation and proliferation [30]. In general macrophages were fully differentiated by day 6.

Gene inserts of fluorescently tagged signaling protein of interest (Syk-mCitrine and BtkPH-mCitrine) were introduced into Murine leukemia virus (MLV)-based vectors. The assembled constructs were used to transfect Plat-E cells using FuGENE following the manufactures protocol. The retroviral supernatant was harvested 48 hours post transfection and used within one week after harvesting. BMMs were plated in the 6-well dish at a density of 1x10^6^ per well. Retroviral supernatants (1x10^7^ virus/mL) were added to the well in the presence of polybrene (10 μg/mL). BMMs were incubated with the virus for 24–48 hours, and then replaced with fresh bone marrow media. These transduced cells were used for following imaging experiments [31].

### Image acquisition and data analysis

TIRF 360 was used to create uniform TIRF illumination by steering the laser at the back-focal plane. The microscope was custom-built based on iMIC system (TILL Photonics, Munich, Germany) with 60x 1.49 oil immersion objective lens (Olympus, Tokyo, Japan), previously described in [29]. BMMs were lifted from culture dish, washed with PBS twice and then dropped onto the SLB surface in the imaging chamber. Cell samples were imaged 3 min after they were placed to the imaging chamber and images were acquired every 5 sec for a total duration of 6 min.

Cell images were processed in Matlab (The MathWorks, Inc., Natick, MA) with customized codes. Two channels were registered using the fiducial data registration method. Multiple-fluorophore beads (TetraSpeck, Invitrogen, CA) were employed for image registration [32]. Individual protein complexes were analyzed with single particle tracking technique. Due to the dynamic movement, some complexes were moving out of the TIRF field. We only tracked molecules that moved within the TIRF field [33]. We imaged at least 3 cells per each condition and performed the tracking and analysis from single cells as the behavior was consistent for all cells imaged under each condition.

## Results and discussion

### IgG-coated SLB for TIRF imaging of FcγR signaling on macrophages

**Fig. 2:**
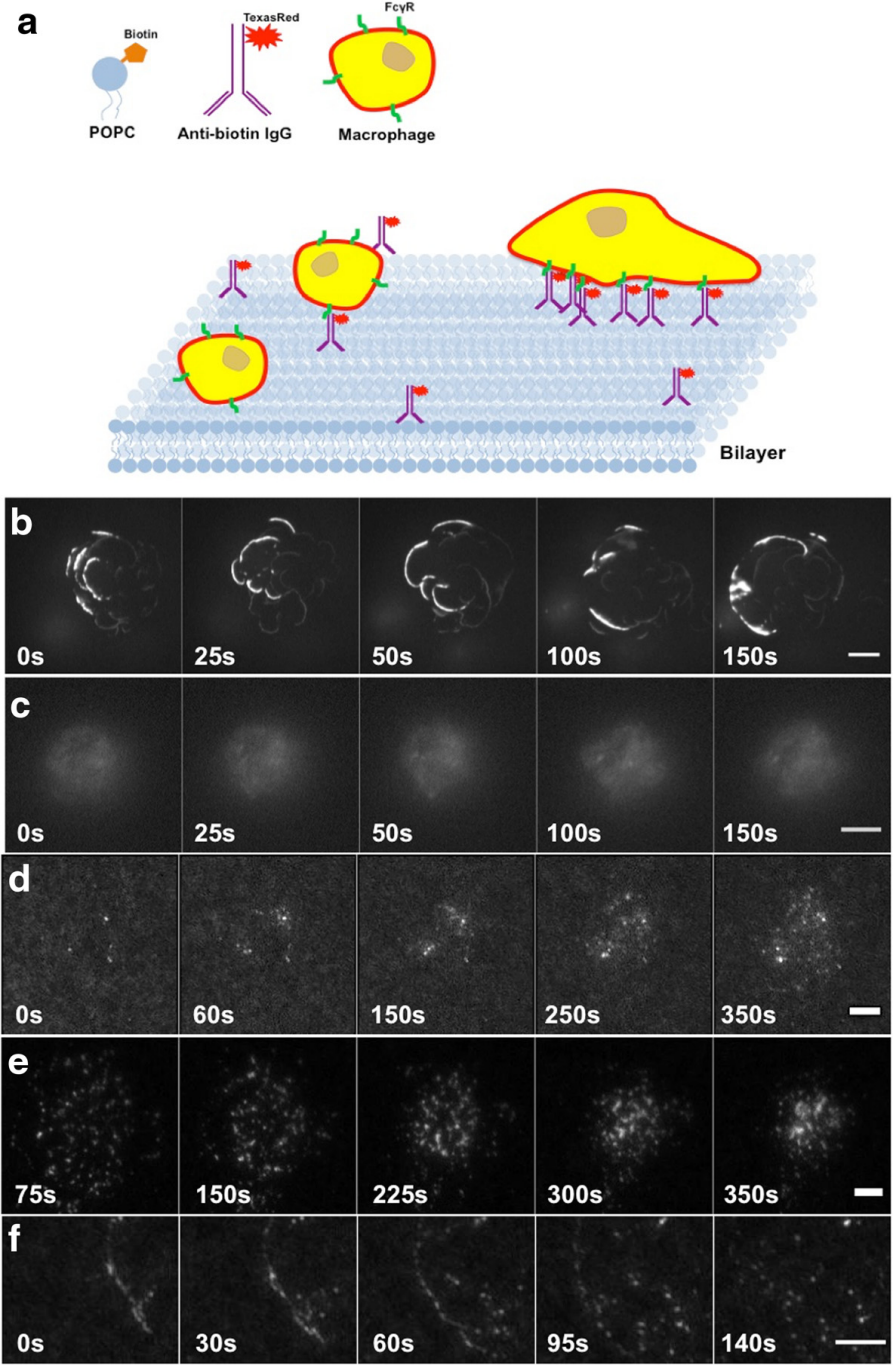
IgG-coated supported lipid bilayer permits selective activation FcγR on macrophages. **a** Cartoon of the supported lipid bilayer (SLB) presentation of IgG via its interaction with Biotin-PE. Bone marrow derived macrophages (BMM) were dropped onto this surface and the plasma membrane in contact with the glass was imaged by total internal reflection fluorescence (TIRF) microscopy. Upon contact with IgG-coated SLB containing 1 % Biotin-PE, BMM labeled with DiI spread and form multiple dynamic pseudopodia, analogous to phagocytosis (phagocytic pseudopod extension). **c** BMM did not engage SLB lacking IgG and simply rolled along the surface. **d-f** Alexa Fluor 594-IgG, allowed imaging of the dynamics of FcγR-IgG complexes during the initial interaction and spreading phase (**d**) during which they formed microclusters that moved with the extending pseudopod. **e** After about 3–5 min (montage starts about 3 min after adding BMM to the SLB), FcγR-IgG microclusters would traffic toward the center of the cell, where they would accumulate. **f** Magnified montage showing the early stages of cell spreading, FcγR-IgG were observed to decorate the advancing pseudopod edge, whereupon they would continue to cluster and eventually begin a retrograde motion toward the center of the cell. Scale bar 10 μm for (**b-c**) and 5 μm for (**d-f**)

We applied the IgG-SLB system to specifically activate FcγR with IgG, while eliminating the incidental activation of other receptors (such as integrins) via interactions with bare glass or serum-coated deposits on glass (Fig. 2a). Previously, IgG-coated glass has been used to study frustrated IgG-mediated phagocytosis; however, macrophages bind to bare glass or serum coated glass indicating \ other receptors are engaged by the glass substrate thereby complicating the study of FcγR. To eliminate activation of other receptors, we developed the SLB system coated with anti-Biotin IgG that recognized Biotin-PE (1 mol %) in the SLB (Fig. 2a). DiI labeled BMMs were placed on IgG-coated SLB and imaged in TIRF (Fig. 2b). We observed that once the macrophage made initial contact with the bilayer, it engaged in very dynamic ruffling through the interactions with the IgG-coated SLB surface (Fig. 2b). The macrophages formed multiple dynamic pseudopods on the IgG-SLB surface (Fig. 2b and Additional file 2). FcγR-IgG microclusters formed at the front of newly formed ruffles at the leading edge of the cell (Fig. 2b). Importantly, macrophages did not interact with SLB lacking IgG, and simply rolled along its surface indicating that the SLB eliminated non-specific interactions with the glass and incidental activation of other receptors (Fig. 2c and Additional file 3). Thus, the IgG-coated SLB allowed for selective activation of FcγRs and enabled precise analysis of FcγR signaling by TIRF microscopy. Furthermore, the mobility of IgG on the SLB afforded a direct comparison of FcγR signaling to the analysis of TCR and BCR, which have been studied using similar systems. In these experiments a monoclonal IgG of unknown subclass was used, but this system could be used to examine differences that arise from interactions of FcγRs with IgGs of different subclasses or glycosylation. A common observation from these data suggests that FcγRs initially cluster and move with the cell’s leading edge and then disengage from the leading edge to undergo retrograde motion toward a central location, reminiscent of TCR and BCR clusters [34–36].

### Dynamic association of Syk with FcγR microclusters

**Fig. 3:**
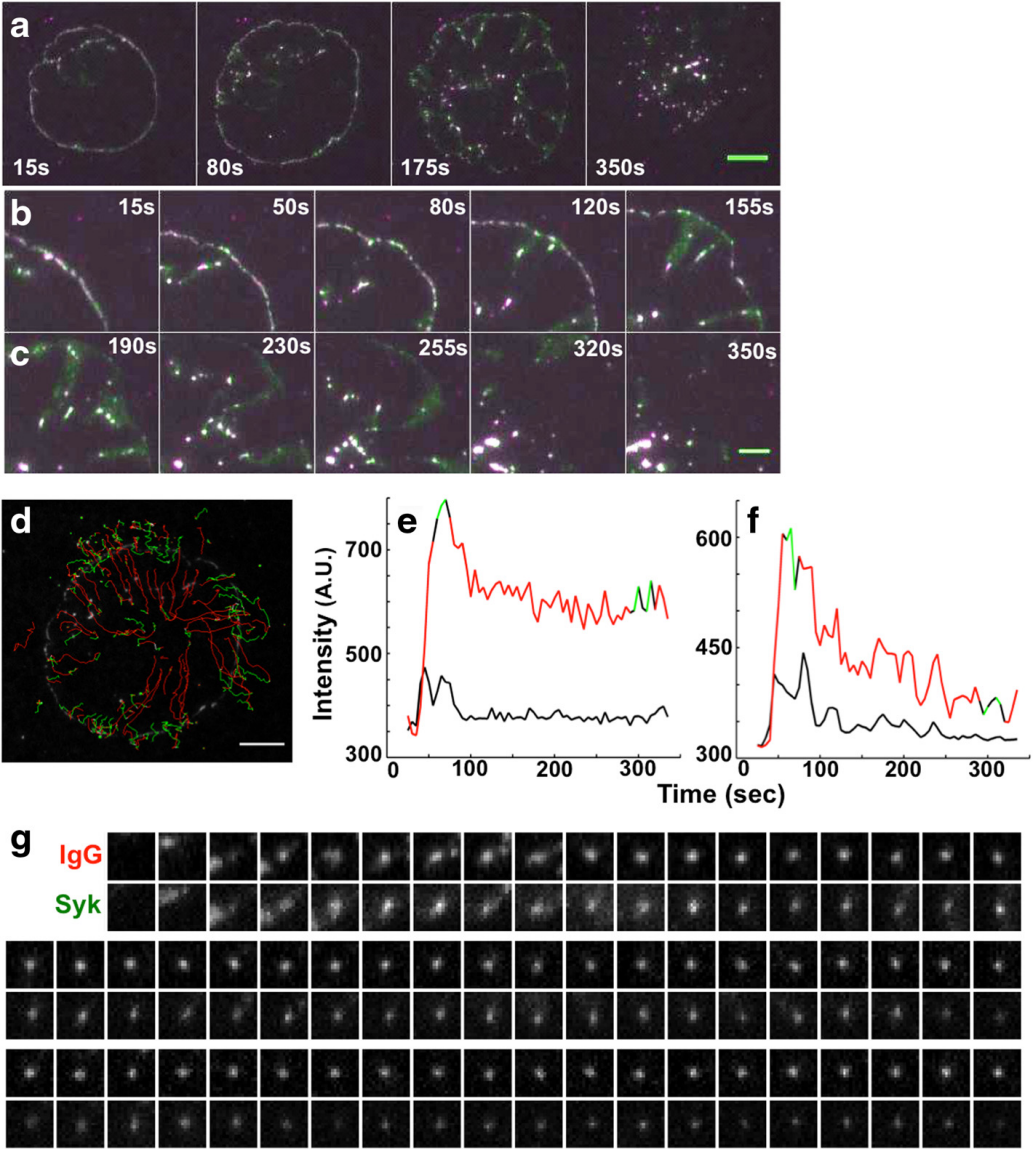
Recruitment of Syk kinase to FcγR-IgG microclusters. **a** Syk (green) associated with FcγR-IgG clusters (magenta) at the leading edge and during retrograde movement of the FcγR-IgG; scale bar 5 μm. B-C) Magnified region from (**a**) shows the association of Syk with FcγR-IgG clusters at the pseudopod leading edge (**b**) and retrograde movement following their detachment from the leading edge (**c**); scale bar 4 μm. **d** Tracking of individual FcγR-IgG microclusters from the cell in (**a**) (green indicates movement away from the cell center and red indicates movement toward the center of the cell); scale bar 10 μm. Intensity of IgG (**e**) and Syk kinase (**f**) associated with a single FcγR-IgG cluster for one track; color-coding indicates directionality of the FcγR-IgG microcluster. The black line indicates the local background signal. These results illustrate the typical behavior of FcγR-IgG microclusters as the they form at the leading edge followed by a static intensity; whereas fluctuations in Syk localization and an overall decrease in signal was observed as the microcluster departed from the leading edge. We performed tracking on one representative cell with a total number of 117 tracks. **g** Montage showing the oscillations in Syk intensity on the FcγR-IgG cluster; the width of the montage panel 2 μm

We imaged the association of Syk with FcγR-IgG microclusters since Syk is the critical kinase for FcγR-mediated phagocytosis and has been demonstrated to promote FcγRs clustering [2, 3]. To image Syk, Syk-mCitrine was introduced into BMMs by retroviral transduction. The Syk-mCitrine expressing cells were dropped onto IgG-coated SLB and imaged by TIRF microscopy (N = 4). Upon cell engagement with the IgG-coated SLB, Syk was recruited to FcγR-IgG complexes at the earliest observable time points (Fig. 3a and Additional file 4). Furthermore, during cell spreading, Syk clusters were detected at the advancing margins of the pseudopod where new FcγR microclusters were forming (Fig. 3b). When the advancing pseudopod reached a maximum diameter, the FcγR microclusters detached from the advancing margin and moved toward the center of the cell (Fig. 3c). This process repeated for about 2–6 minutes while Syk and FcγR microclusters accumulated in the center of the cell (Fig. 3c). We performed tracking of individual FcγR-IgG complexes and plotted them over the cell image (Fig. 3d). These tracks were color coded for directionality – objects moving away from the center of the cell (green) and toward the center of the cell (red) (Fig. 3d). In these tracks, we observed oscillations in the association of Syk with FcγR microclusters (Fig. 3e-g). By tracking individual FcγR-IgG microclusters and quantifying the intensity of IgG and Syk, we could see that IgG intensity rose and leveled-off as the microcluster formed, which was followed by retrograde flow toward the center of the cell. Syk followed a similar trend but its intensity oscillated (Fig. 3e-g). This oscillation could be observed when montages were made of single FcγR-IgG microclusters (Fig. 3f and g), despite a constant fluorescence signal from IgG. Thus, we conclude that turnover of Syk potentially mediated by repeated rounds of phosphorylation and dephosphorylation are part of the typical FcγR signaling mechanism [2].

### Dynamics of actin and PI(3,4,5)P_3_ relative to IgG-FcγR complexes

**Fig. 4:**
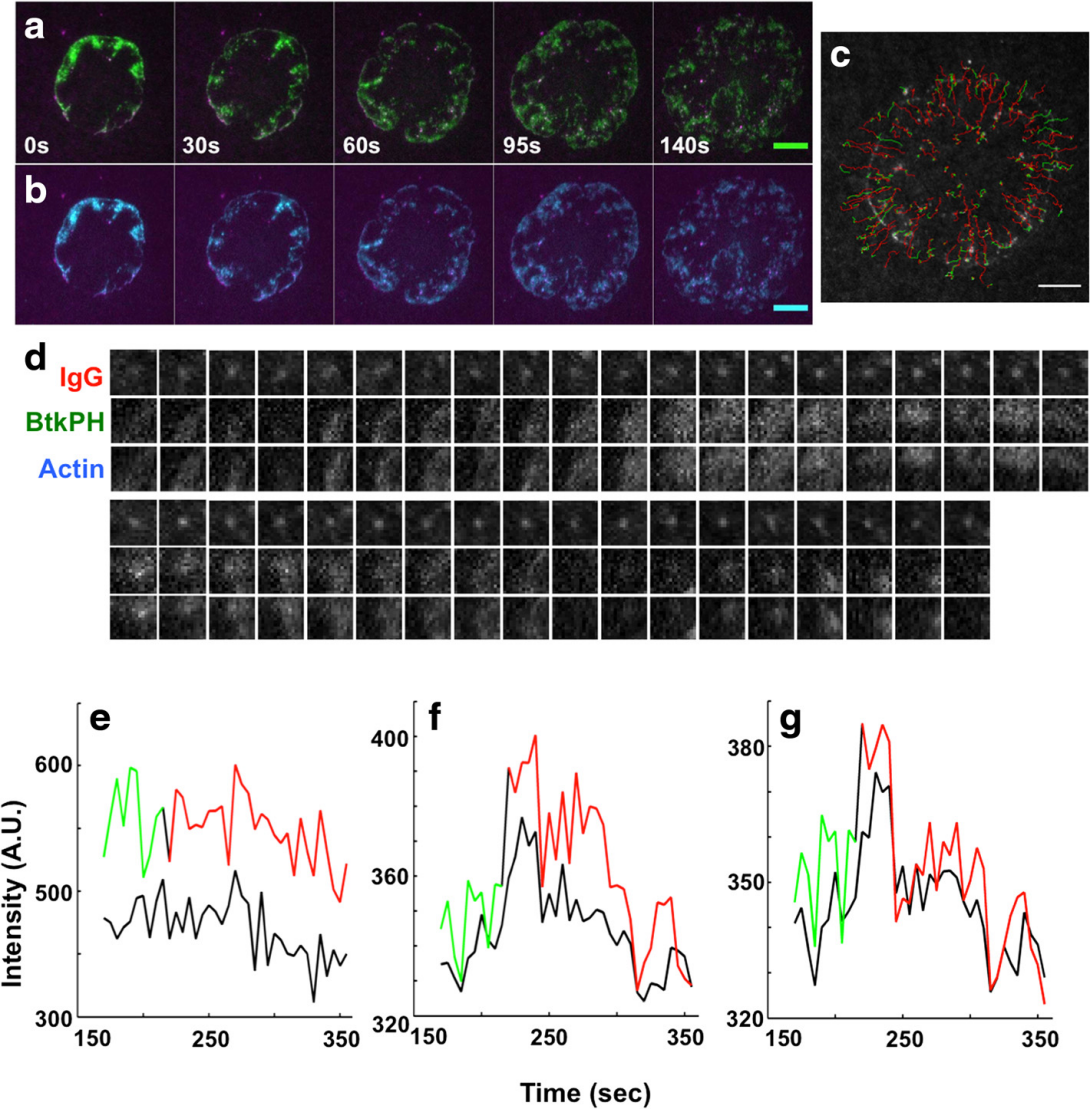
PI(3,4,5)P_3_ and actin display coordinated but diffuse distributions around FcγR-IgG clusters. BtkPH (green, **a**) and actin (cyan, **b**) were distributed along the leading edge of the advancing pseudopod, near or behind clustered FcγR-IgG (magenta). FcγR-IgG clusters departed from the leading edge have both BtkPH and actin near, but not coincident with them; scale bar 10 μm. **c** FcγR-IgG microclusters were tracked, and color-coded, green – outward, red – inward; the tracking was done on one cell with a total number of 172 tracks; scale bar 10 μm. **d** Analysis of single IgG clusters showed diffuse recruitment of BtkPH consistent with PI(3,4,5)P_3_‘s ability to diffuse in the membrane and with actin polymerization in areas rich in PI(3,4,5)P_3_; the width of the montage panel 2 μm. **e-f** Intensity traces from the track in (**d**) indicated that while the IgG signal remained constant in the cluster (**e**), the associated BtkPH (**f**) and actin (**g**) signals underwent coincident transients that were correlated to the motion of the FcγR-IgG clusters; color coding is the same as in (**c**). The black line indicates the local background signal

To define the relationship between FcγR-IgG microclusters, PI3K activity and the actin cytoskeleton, we generated BMM expressing BtkPH fused with mCitrine and lifeact-GFP and imaged them on the IgG-SLB (Fig. 4a-b and Additional files 5 and 6). At early time points, BtkPH was recruited to the leading edge of the cell and had the highest fluorescent signal near FcγR microclusters (Fig. 4a, d, N = 3 cells). Importantly BtkPH fluorescence was diffusely localized around the FcγR microclusters, which was distinct from the pattern we observed in experiments with Syk (Fig. 4d). We interpret this diffuse pattern of PI(3,4,5)P_3_ away from FcγR-IgG microclusters as an important mechanism for propagation of signals laterally in the membrane. When comparing actin location and dynamics with BtkPH (Fig. 4a-b), we observed a striking similarity. This observation suggests that both actin and BtkPH are recruited to sites of activated FcγR microclusters during frustrated phagocytosis. We performed single particle tracking on FcγR-IgG (Fig. 4c-d) and corresponding to it BtkPH and actin (Fig. 4d). To highlight the change in direction of tracked objects, we color-coded outward and inward object movements with green and red, respectively (Fig. 4). By tracking and measuring the fluorescence intensity of FcγR microclusters, BtkPH and actin, we found a strong correlation between actin and BtkPH (Fig. 4d-g). This observation is consistent with previous studies indicating the requirement of PI3K activation mediate actin-dependent membrane protrusion and closure of phagosomes around large particles [12, 15, 37, 38]. Furthermore, it illustrates PI(3,4,5)P_3_ distributions radiate away from the FcγR-IgG microcluster where they coordinate the actin cytoskeleton via other mediators such as Rac and Cdc42 [4, 10]. Thus, PI(3,4,5)P_3_ radiates from FcγR microclusters and recruits additional signaling molecules that either promote interaction with the FcγR microcluster or simply localize key proteins to the phagosome membrane.

## Conclusion

In this work we demonstrated that like the TCR, BCR, and FcεR, FcγR on macrophages form microclusters that are transported into a synapse-like structure. In addition, this work provides a powerful system in which SLB presentation of IgG can be used to specifically activate FcγR on the surface of macrophages without incidental activation of other receptors by the glass surface. Using this system, we were able to observe FcγR microclustering at the pseudopod edge of macrophages engaged in frustrated phagocytosis, followed by the release of these receptors from the leading edge and their subsequent retrograde transport. By expressing fluorescent proteins in these cells and tracking the motions of FcγR-IgG microclusters, we were able to make two new observations. First, we found that as expected, Syk localized to the FcγR-IgG microcluster where it oscillated on and off, suggesting multiple rounds of phosphorylation of the FcγR. Second, we observed local PI(3,4,5)P_3_ was produced proximal to FcγR-IgG microclusters and this localization corresponded well with the localization of actin at the leading edge of the pseudopod and to a lesser extent on FcγR microclusters that have departed from the leading edge. In comparing this system to other imaging experiments that track the localization of downstream signaling components to IgG coated beads and erythrocytes, we note similar behaviors – Syk remains associated with the FcγR at all stages as observed for IgG bead phagosomes [11], and that PI(3,4,5)P_3_ is coordinated with actin on membrane that remains contiguous with the plasma membrane [39]. Together, these observations indicate that there are many parallels across immunoreceptor signaling and that microclustering and actin-mediated transport of these receptors is a common theme. Furthermore, this approach can be used to address key questions regarding FcγR activation, deactivation and signal propagation.

### Ethics statement

This research does not involve human subjects, human materials. All animal research protocols for this work were reviewed and approved by the IACUC committee at South Dakota State University.

## Additional files

Additional file 1: Video 1. FRAP of the Bodipy labeled bilayer. The mobility of SLB and photobleaching effect were checked with a fluorescence recovery after photobleaching (FRAP) microscopy. SLB was labeled with Bodipy (5 μg/ml) and a region was bleached and the recovery was recorded. Scale bar 10 μm. (AVI 857 kb) Additional file 2: Video 2. DiI labeled BMM engage and spread on IgG-SLB. PC/PE-Biotin in the SLB was labeled with anti-Biotin IgG. Macrophages dropped onto this surface actively engaged in frustrated phagocytosis. Scale bar 10 μm. (AVI 310 kb) Additional file 3: Video 3. DiI-labeled BMM do not interact with SLB in the absence of IgG and simply roll alone the surface. Scale bar 10 μm. (AVI 524 kb) Additional file 4: Video 4. FcγR-IgG (magenta) microcluster formation and Syk recruitment (green). Syk associated with FcγR-IgG microclusters at the leading edge during spreading phase and to a lesser extent following accumulation of FcγR-IgG microclusters in the center of the cell. Oscillations were observed for Syk kinase as it was recruited to the FcγR-IgG microclusters. Scale bar 10 μm. (AVI 628 kb) Additional file 5: Video 5. Dynamics of PI(3,4,5)P_3_ was tracked by imaging BtkPH (green) relative to FcγR-IgG (magenta) microclusters. BtkPH association with clustered receptors indicates lateral propagation of signals. Scale bar 10 μm. (AVI 1549 kb) Additional file 6: Video 6. Dynamics of actin (cyan) in relation to FcγR-IgG (magenta) microclusters. These data are for the same cell as shown in Additional file 5. Fluorescent signal from Actin follow very similar patterns and dynamics as BtkPH. Scale bar 10 μm. (AVI 1617 kb)

## Abbreviations

APC: antigen-presenting cell
BCR: B-cell receptor
Biotin-PE: 1-oleoyl-2-(12-biotinyl(aminododecanoyl))-*sn*-glycero-3-phosphoethanolamine
BtkPH: Bruton’s tyrosine kinase PH domain
cSMAC: center of SMAC
dSMAC: distal SMAC
FRAP: fluorescent recovery after photobleaching
Fc: fragment crystalizable region
FcγR: Fcγ receptor
GEF: Guanine nucleotide exchange factor
IgG: immunoglobulin G
IS: immunological synapses
ITAM: immunoreceptor tyrosine-based activation motif
MCSF: macrophage colony-stimulating factor
PC: l-Palmitoyl-2-oleoyl-sn-glycero-3-phosphocholine
PI3K: phosphatidylinositol 3-kinase
PI(4,5)P_2_: phosphatidylinositol 4,5-bisphosphate
PI(3,4,5)P_3_: phosphatidylinositol (3,4,5)-trisphosphate
SLB: supported lipid bilayer
Syk: spleen tyrosine kinase
SMAC: supramolecular activation cluster
TCR: T-cell receptor
TIRF microscopy: total internal reflection microscopy
